# A Carboxy-Terminal Trimerization Domain Stabilizes Conformational Epitopes on the Stalk Domain of Soluble Recombinant Hemagglutinin Substrates

**DOI:** 10.1371/journal.pone.0043603

**Published:** 2012-08-23

**Authors:** Florian Krammer, Irina Margine, Gene S. Tan, Natalie Pica, Jens C. Krause, Peter Palese

**Affiliations:** 1 Department of Microbiology, Mount Sinai School of Medicine, New York, New York, United States of America; 2 Department of Medicine, Mount Sinai School of Medicine, New York, New York, United States of America; 3 Graduate School of Biological Sciences, Mount Sinai School of Medicine, New York, New York, United States of America; University of Massachusetts Medical Center, United States of America

## Abstract

Recently, a new class of broadly neutralizing anti-influenza virus antibodies that target the stalk domain of the viral hemagglutinin was discovered. As such, induction, isolation, characterization, and quantification of these novel antibodies has become an area of intense research and great interest. Since most of these antibodies bind to conformational epitopes, the structural integrity of hemagglutinin substrates for the detection and quantification of these antibodies is of high importance. Here we evaluate the binding of these antibodies to soluble, secreted hemagglutinins with or without a carboxy-terminal trimerization domain based on the natural trimerization domain of T4 phage fibritin. The lack of such a domain completely abolishes binding to group 1 hemagglutinins and also affects binding to group 2 hemagglutinins. Additionally, the presence of a trimerization domain positively influences soluble hemagglutinin stability during expression and purification. Our findings suggest that a carboxy-terminal trimerization domain is a necessary requirement for the structural integrity of stalk epitopes on recombinant soluble influenza virus hemagglutinin.

## Introduction

Hemagglutinin (HA) exists as a glycoprotein trimer on the surface of the influenza virion. Each monomer is initially expressed as HA0, and is subsequently cleaved by host proteases into HA1 and HA2 subunits, which are linked via a disulfide bond. So far, 17 antigenically distinct influenza A HA subtypes have been described and they are further categorized as either group 1 or group 2 hemagglutinins (group 1: H1, H2, H5, H6, H8, H9, H11, H12, H13, H16 and H17 viruses; group 2: H3, H4, H7, H10, H14, and H15 viruses) [1], [2].

HA can be functionally divided into two domains, the globular head and the stalk. The head region contains the receptor-binding site that modulates the ability of the virus to bind to host substrates. Antibodies directed towards this region can block receptor binding and are known to be neutralizing. The stalk domain, which makes up the majority of the amino acid sequence of the HA molecule, mediates virus fusion and uncoating by virtue of the fusion peptide that is located in this domain. Following binding to sialylated host receptors, the virus is internalized by endocytosis. The endosome is then acidified, inducing a conformational change in the HA that facilitates the union of host and viral membranes so that the viral genome can be released into the cytoplasm for subsequent replication, transcription and translation [1]. Recently, a new class of neutralizing antibodies against the stalk of the influenza virus HA has been discovered [3]–[11] and they are thought to block this fusion function.

Because of the importance of HA-directed antibodies in preventing influenza virus infection, recombinant HA is a valuable reagent for influenza virus research and the vaccine industry. Recombinant HA is used to assess sero-conversion of vaccinees and experimental animals, to measure binding kinetics of monoclonal antibodies, or as standard for the quantification of the HA content of vaccines. There are also attempts to use baculoviral-, mammalian- or bacterial-expressed HAs as human or veterinarian vaccines [12]–[17]. Despite the widespread use of recombinant HA in the field, expression and purification methods for HA vary greatly. Full length or truncation mutants have been expressed using a variety of expression systems [18]–[21]. The biochemical and antigenic characteristics of the produced reagents are therefore influenced by the sequence of the HA expression construct, the production system and the purification method.

Much excitement has surrounded the discovery of antibodies that bind the stalk domain of the HA molecule. Most globular head antibodies are strain-specific because of antigenic drift in the hypervariable loops of this domain. In contrast, the stalk domain is highly conserved, and antibodies directed against the stalk are more likely to be cross-reactive, even between subtypes [3]–[8], [10], [11], [22]. Several of these novel neutralizing antibodies have been shown to bind conformational epitopes that are present in the pre-fusion conformation of the HA [4], [5], [7], [22]. Reagents for the detection and quantification of this new class of antibodies require structural integrity of the stalk domain in order to preserve these conformational epitopes. We hypothesized that soluble HAs would exhibit impaired folding of the stalk domain in the absence of a membrane that normally orders their trimeric structure, as on the surface of the influenza virion. We rationalized that the inclusion of a trimerization domain would allow for proper trimeric association between HA monomers and, by doing this, would provide for proper folding of the stalk. To test this theory, we fused the sequence of the extracellular domain of the HA to a short linker region, along with a thrombin cleavage site, a natural trimerization domain from T4 phage fibritin, and a hexahistidine purification tag [23]. We chose to produce and test secreted recombinant HA with and without a carboxy-terminal (C-terminal) trimerization domain in the baculoviral expression system, a system that is easy to establish and widely used in influenza research laboratories. We then assessed the binding of stalk-reactive antibodies to group 1 and group 2 HAs with or without a trimerization domain. Our findings confirm the importance of stabilizing the stalk structure in recombinant HAs for the detection of stalk-reactive antibodies and provide insight into the fragile nature of conformational stalk epitopes.

## Materials and Methods

### Cells

Sf9 insect cells (ATCC # CRL-1711) were grown in TMN-FH medium (Gemini Bio-Products) supplemented with 10% FBS (Atlanta Biologicals), 0.1% Pluronic F68 (Sigma) and a Penicillin-Streptomycin antibiotic (Gibco) mixture. BTI-TN-5B1-4 cells (High Five - Vienna Institute of Biotechnology subclone [24]) were grown in HyClone SFX serum free medium (Fisher Scientific) supplemented with Penicillin-Streptomycin antibiotic mixture (Gibco).

### Cloning and Recombinant Baculovirus Generation

**Figure 1:**
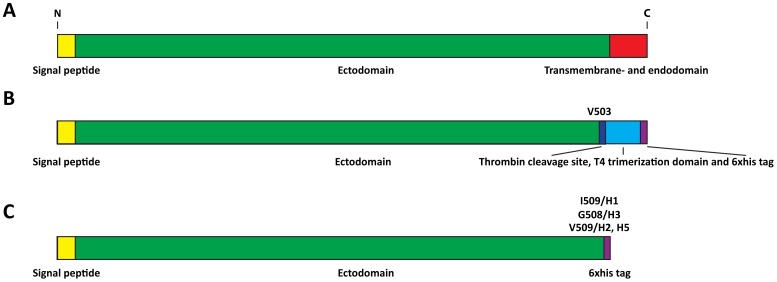
Schematic of wild type HA and expression constructs. **A** Uncleaved full length influenza virus hemagglutinin. The signal peptide is indicated in yellow, the HA ectodomain is indicated in green and transmembrane- and endodomain are held in red. **B** Expression construct with trimerization domain. The transmembrane- and endodomain was swapped with a thrombin cleavage site (dark blue), a T4 trimerization domain (light blue) and a hexahistidine tag (6xhis tag, purple) at position V503 (H3 numbering). **C** Expression construct without trimerization domain. The transmembrane- and endodomain was swapped with a hexahistidine tag (6xhis tag, purple) at amino acid position 509 (H1, H2 and H5) or 508 (H3) respectively (H3 numbering).

Sequences coding for HAs of H1 strains A/Puerto Rico/8/34 (PR8), A/California/04/09 (Cal09), H2 strain A/Japan/305/57 (JAP57), H3 strains A/Hong Kong/1/68 (HK68), A/Wisconsin/67/05 (Wisc05) and H5 strain A/Viet Nam/1203/04 (VN04 - with removed polybasic cleavage site [25]) were amplified from pCAGGS plasmids by polymerase chain reaction and cloned into a modified pFastBac vector (Invitrogen) using *Bam*HI or *Stu*I and *Not*I restriction endonucleases (NEB). Primer sequences are available upon request. Two sets of constructs, HA without and with trimerization domain, were cloned: HA constructs without trimerization domain were designed so that the C-terminal transmembrane- and endodomain of the HA were replaced with a hexahistidine-tag (HA sequence ends with I509 for H1, V509 for H2 and H5 and G508 for H3; H3 numbering); the other set of constructs, HA with a trimerization domain, also lack the C-terminal transmembrane- and endodomains (HA sequence ends with V503 - H3 numbering) but include a thrombin cleavage site and a T4 foldon trimerization domain [26] in addition to the C-terminal hexahistidine-tag (Figure 1). Generated recombinant pFastBac clones were transformed into DH10Bac bacteria (Invitrogen) according to the manufacturer's instructions and recombinant bacmids were prepared with a PureLink Plasmid Filter Midiprep kit (Invitrogen). Recombinant bacmids were transformed into Sf9 cells using Cellfectin II (Invitrogen) for rescue of recombinant baculovirus. All sequences were confirmed by Sanger sequencing.

### Protein Expression, Purification and Characterization

Baculovirus was amplified in Sf9 cells to a passage 3 stock and then used to infect BTI-TN-5B1-4 (High Five) cells at 1×10^6^ cells/ml in HyClone SFX serum free media (Fisher Scientific) at a multiplicity of infection of 10. Expression was carried out in 1000 ml shaker flasks for 96 hours at 28°C. After 96 hours, supernatants were cleared by low speed centrifugation (5000 g, 4°C, 20 min) and incubated with Ni-NTA (Qiagen) resin (3 ml slurry for 250 ml of culture supernatant) for two hours at room temperature (RT). The resin-supernatant mixture was then passed over 10 ml polypropylene columns (Qiagen). The retained resin was washed four times with 15 ml of washing buffer (50 mM Na_2_HCO_3_, 300 mM NaCl, 20 mM imidazole, pH 8) and protein was eluted with elution buffer (50 mM Na_2_HCO_3_, 300 mM NaCl, 300 mM imidazole, pH 8). The eluate was concentrated using Amicon Ultracell (Millipore) centrifugation units with a cut-off of 30 kDa and buffer was changed to phosphate buffered saline (PBS) of pH 7.4. Protein concentration was quantified using Quickstart Bradford Dye Reagent (Bio-Rad) with a bovine serum albumin standard curve. Protein purity, integrity and identity was assessed by sodium dodecyl sulfate polyacrylamide gel electrophoresis (SDS-PAGE) (4–20% polyacrylamide - Mini PROTEAN TGX gels, Bio-Rad), Coomassie staining and Western blot or enzyme linked immunosorbent assay (ELISA). Extent of trimerization and/or multimerization was tested by crosslinking of HA with bis-[sulfosuccinimidyl]suberate (BS^3^ - Fisher Scientific) according to the manufacturer's recommendations. Briefly, 3 µg of HA were incubated in 30 µl of PBS in the presence of a 25 fold molar excess of BS^3^ crosslinker. The mixture was incubated at RT for 30 minutes and then BS^3^ was quenched by adding 1 M Tris-HCl buffer (pH 8) to a final concentration of 50 mM. Subsequently SDS-PAGE and/or Western blot analysis with a mouse anti-his primary antibody (Sigma) and anti-mouse horseradish peroxidase (Santa Cruz Biotechnology) or alkaline phosphatase (Santa Cruz Biotechnology) conjugated secondary antibody was performed.

### Enzyme Linked Immunosorbent Assay

Immunolon 4HBX (Fisher Scientific) plates were coated with recombinant HA with and without trimerization domain at a concentration of 5 µg/ml in coating buffer (0.1 M Na_2_CO_3_/NaHCO_3_, pH 9.2, 50 µl/well) overnight at 4°C. The plates were then blocked for one hour at RT with PBS (pH 7.4) containing 1% Tween 20 (TBPS) and 3% non-fat dry milk powder. After blocking, plates were washed once with TPBS and then incubated with three fold dilutions of monoclonal antibody or sera (100 µl per well in TPBS with 1% milk powder – monoclonal antibody starting concentration 30 µg/ml; 1∶100 dilution for sera) for one hour at RT. Plates were then washed trice with 100 µl of TPBS and incubated for another hour at RT with horse radish peroxidase conjugated anti-mouse IgG (Santa Cruz Biotechnology) or anti-human Fab secondary antibody (Sigma) at a dilution of 1∶3000 (50 µl per well). After three more washes, plates were developed using SigmaFAST OPD substrate (Sigma) (100 µl/well), stopped with 3 M HCl (50 µl/well) and read at an absorption of 490 nm on a Synergy 4 (BioTek) plate reader. The obtained read-out was background subtracted with values from secondary antibody-only incubated wells.

Cal09 and PR8 HAs were probed with mouse monoclonal antibodies C179 [11], 6F12 [22], KB2 [27], BD3, IB11 and GG3 (the latter three unpublished) and human Fab fragment from CR6261 [4]. Additionally, mouse monoclonals PY102 (PR8) [28] and 7B2 (Cal09) [22] and PR8 and Cal09 mouse antisera were used for strain specific detection. JAP57 HA was detected using mouse monoclonal antibody C179, mouse anti-sera against JAP57 VLPs [29] and a human Fab fragment from CR6261 or human anti-H2 IgG 8F8 [30]. HK68 and Wisc05 HAs were probed using mouse monoclonal 12D1 [8], mouse H3 antisera, human Fab from CR8020 [5] and strain specific antibody XY102 (HK68) [31]. Finally, VN04 was probed using mouse monoclonals C179, KB2, GG3, IB11, BD3 and mAb#8 [32] as well as VN04 mouse antisera and a human Fab fragment from CR6261.

For stability studies, HA from PR8 virus with trimerization domain was stored at 4°C for 60 days, or at −80°C and went through one (standard), two, three or four freeze-thaw cycles. Stability of head versus stalk binding antibodies was compared using PY102 and C179 monoclonal antibodies. Antibody-HA combinations in ELISA were done in triplicates except for stability studies where duplicates were used.

## Results

### A C-terminal Trimerization Domain Stabilizes HAs and Induces Trimer Formation

**Figure 2:**
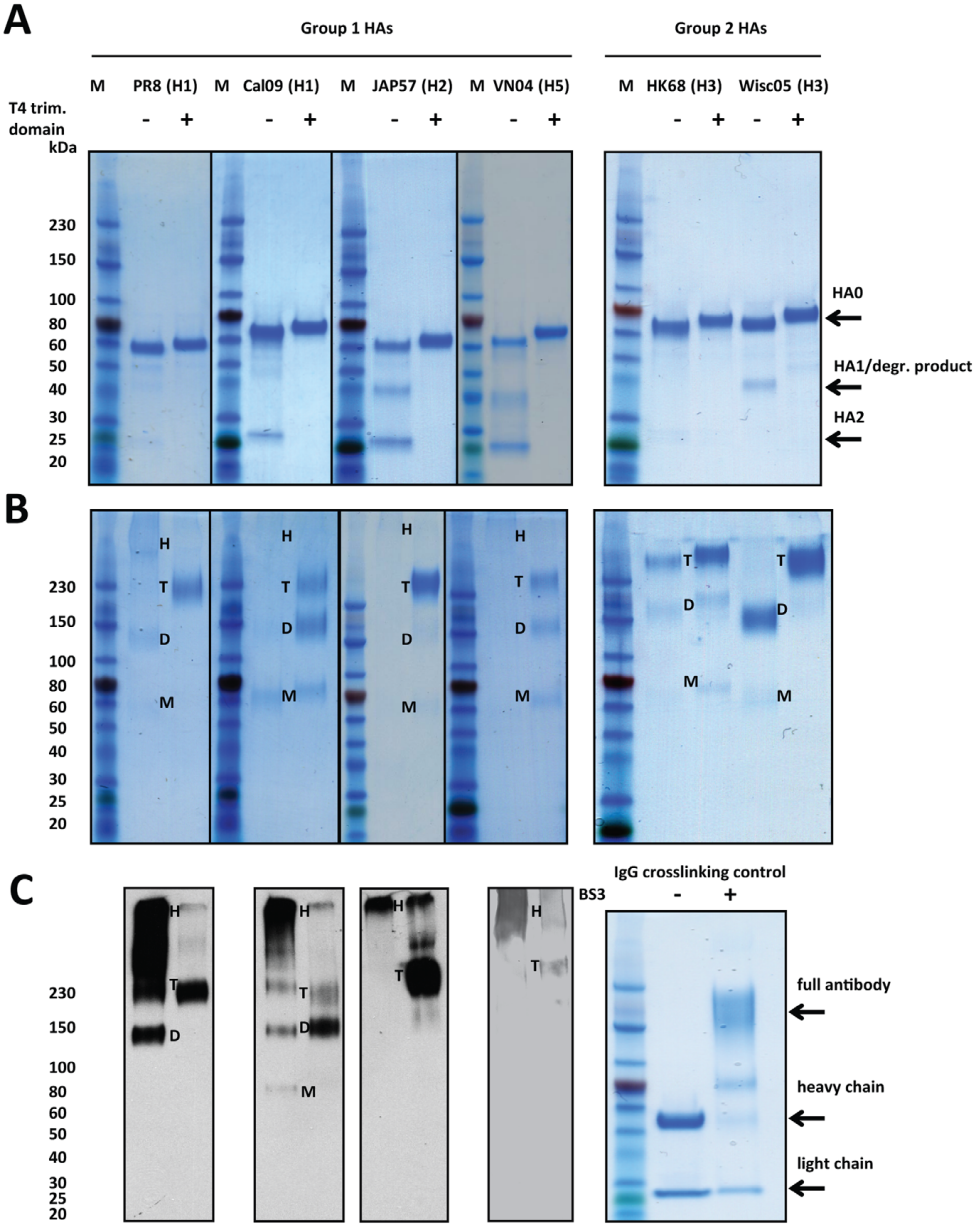
Introduction of a trimerization domain influences stability and formation of oligomers in recombinant HAs. **A** Analysis of recombinant HAs with and without trimerization domain by reducing, denaturing SDS-PAGE. Recombinant HAs that are expressed with trimerization domain (+) show higher stability than HAs expressed without (−). Uncleaved HA (HA0) and cleavage products (HA1/degr. product; HA2) are indicated by arrows. **B** Reducing, denaturing SDS-PAGE analysis of crosslinked HAs. Different species of HA are indicated in the blot. High molecular multimers are indicated by H, trimers by T, dimers by D and monomers by M. **C** Left panel: Western blot analysis of reduced, denatured and cross-linked group 1 HAs from B probed with a anti-hexahistidine-tag antibody. Right panel: Cross-linking control (IgG) with BS^3^ analyzed on a SDS-PAGE. Different species (full antibody, heavy chain, light chain) are indicated by arrows. Molecular weights of the marker bands are indicated on the left of each panel.

We expressed the extracellular domain of various group 1 and group 2 HAs in soluble form with or without a C-terminal T4 phage trimerization domain (Figure 1) in the baculoviral expression system. Proteins were harvested 96 hours post infection and purified via a C-terminal hexahistidine-tag using a Ni-NTA column. Purified protein was concentrated using ultrafiltration spin columns, assessed for protein integrity and impurities by SDS-PAGE and Coomassie staining and quantified with Bradford reagent. Based on the amino acid sequence and the fact that baculovirus expressed full length HAs without polybasic cleavage site are usually uncleaved [29], [33], the extracellular domain of HA would have an expected molecular mass of approximately 60 kDa per monomer (or 180 kDa per trimer) without taking glycosylation into account. Cal09 (H1), JAP57 (H2) and VN04 (H5 without polybasic cleavage site) HA without trimerization domain seemed to be partially cleaved into HA1 and HA2 as indicated by the presence of bands at approximately 40 kDa (HA1) and 25 kDa (HA2) in addition to the uncleaved HA band at 60 kDa (HA0). Based on the exclusive presence of a 60 kDa band for Cal09, JAP57 and VN04 HAs with trimerization domains in the non-reducing, denaturing SDS-PAGE, we can assume that these proteins are expressed mostly as an uncleaved HA0 (Figure 2A). Additionally, preparations of Wisc05 (H3) HA without a trimerization domain showed a degradation product at 40 kDa that was reactive when probed with an anti-stalk antibody (12D1) (data not shown). We therefore believe that this species was a product of non-specific cleavage. Wisc05 HA with trimerization domain however appeared only as an HA0 band (Figure 2A). PR8 and HK68 HA appeared to be very stable (present as HA0) even in the absence of a trimerization domain and therefore stability was not further improved.

We were also interested in assessing whether HAs without trimerization domain would form trimers or would stay in monomeric conformation as recently reported [14]. We crosslinked HAs with and without T4 trimerization domain using BS^3^, a hydrophilic 11 Ångstrom chemical crosslinker that was recently used to show trimerization for HAs [14]. After crosslinking, samples were diluted in a reducing, denaturing loading dye and resolved on a reducing, denaturing SDS-PAGE gel. We found that group 1 HAs without trimerization domain formed high molecular weight oligomers that barely ran into the running gel and were mostly retained in the stacking gel (Figure 2B and C). High molecular weight oligomerization was previously described for bacterial expressed H1 and H5 HA1, and is likely mediated by an oligomerization signal in the N-terminus of the HA [18], [34]. The strongest phenotype was detected for VN04 and JAP57; other group 1 HAs also formed additional trimers (approximately 230 kDa), dimers (130 to 150 kDa) and monomers (60 kDa) (Figure 2B and C). Group 1 HAs with trimerization domain however formed mostly trimers that ran at approximately 230 kD on the SDS-PAGE gel and formed a defined band in the running gel. However, they also formed dimers (approximately 130 to 150 kDa, strongest for Cal09) and monomers (60 kDa). Group 2 HAs behaved differently - HK68 HA formed predominantly trimers and to some degree dimers regardless of the presence of a trimerization domain. Wisc05 HA showed mainly dimerization in the absence of a trimerization domain, while HA with the T4 domain was mostly trimerized.

### A C-terminal Trimerization Domain Strongly Enhances Binding of Stalk-reactive Antibodies to HA Substrates

**Figure 3:**
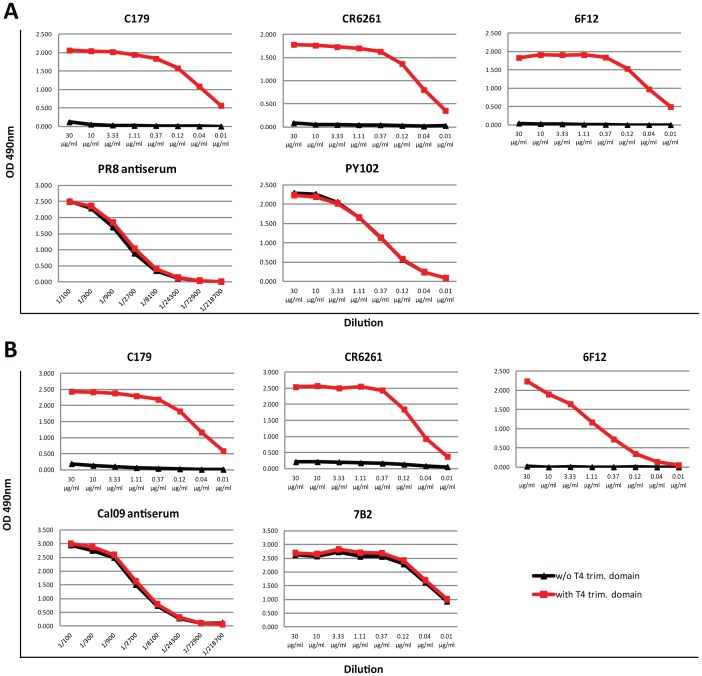
Binding of stalk-reactive antibodies to recombinant PR8 (H1) and Cal09 (H1) HAs. **A** Binding of stalk-reactive antibodies C179, CR6261 and 6F12 and head-reactive antibody PY102 and PR8 antiserum to recombinant soluble PR8 HA without (w/o T4 trim. domain, black lines) or with (w/T4 trim. domain, red line) trimerization domain. **B** Binding of stalk-reactive antibodies C179, CR6261 and 6F12 and head-reactive antibody 7B2 and Cal09 antiserum to recombinant soluble Cal09 HA without (w/o T4 trim. domain, black lines) or with (w/T4 trim. domain, red line) trimerization domain.

Several broadly neutralizing antibodies that react with the stalk domain of influenza virus HA have been reported recently. We tested the reactivity of a panel of such broadly reactive, neutralizing antibodies to our HA constructs in order to determine differential binding of these antibodies to HA substrates with and without trimerization domain. We used well-defined stalk antibodies such as mouse mAb C179, human mAb CR6261 (both group 1 specific), and human mAb CR8020 (group 2 specific) along with antibodies generated by our laboratory (mouse mAb 6F12, group 1 specific [22]), and 12D1, group 2 specific). We also tested four stalk-reactive antibodies, KB2, BD3, GG3 and IB11 (the latter three unpublished), that were recently isolated and characterized by our group to have reactivity to both H1 and H5 HAs. As a control, we used strain specific antibodies that are known to bind to the globular head domain of HA. As additional controls, we tested sera of mice that have been sub-lethally infected with influenza virus strains (PR8, Cal09, H3, VN04) or vaccinated with VLPs (JAP57). Antibodies C179, CR6261 and 6F12 showed a strong binding phenotype to both H1 HAs that were tested (Cal09 and PR8). It is of note that they bound exclusively to HAs that had a trimerization domain (Figure 3A and B); no binding was observed to HAs without a trimerization domain. Similar binding characteristics were seen with the four other stalk-reactive broadly neutralizing H1–H5 antibodies (data not shown). In contrast, head-specific antibodies, such as 7B2 (Cal09) and PY102 (PR8), reacted with HAs irrespective of the expression of a trimerization domain and these findings were confirmed using sera from Cal09 or PR8 infected animals (Figure 3A and B).

**Figure 4:**
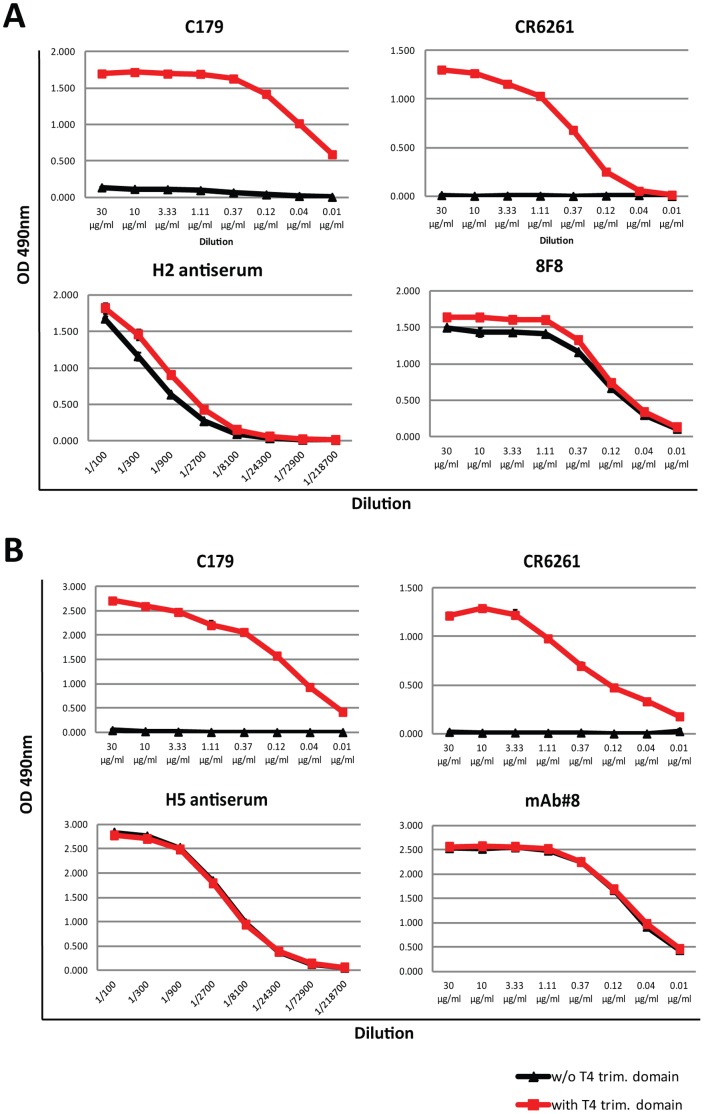
Binding of stalk-reactive antibodies to recombinant JAP57 (H2) and VN04 (H5) HAs. **A** Binding of stalk-reactive antibodies C179 and CR6261 and head-reactive antibody 8F8 and H2 antiserum to recombinant soluble JAP57 HA without (w/o T4 trim. domain, black lines) or with (w/T4 trim. domain, red line) trimerization domain. **B** Binding of stalk-reactive antibodies C179 and CR6261 and head-reactive antibody mAb#8 and H5 antiserum to recombinant soluble VN04 HA without (w/o T4 trim. domain, black lines) or with (w/T4 trim. domain, red line) trimerization domain.

This effect is not specific to the H1 subtype - when testing the binding of C179 and CR6261 to JAP57 (H2) and VN04 (H5) HAs with and without a trimerization domain, a similar phenotype was observed, where these antibodies only reacted with trimerized forms of the protein (Figure 4A and B). The same result was seen when reactivity of the four H1–H5 antibodies was assessed (data not shown). As expected, head-specific antibodies 8F8 (JAP57) and mAb#8 (VN04) or polyclonal anti-H2 or anti-H5 sera recognized both forms of HA equally well. The small difference in binding of 8F8 and JAP57 antisera seen in Figure 4A might stem from slight variations in protein concentration used to coat the ELISA plates.

**Figure 5:**
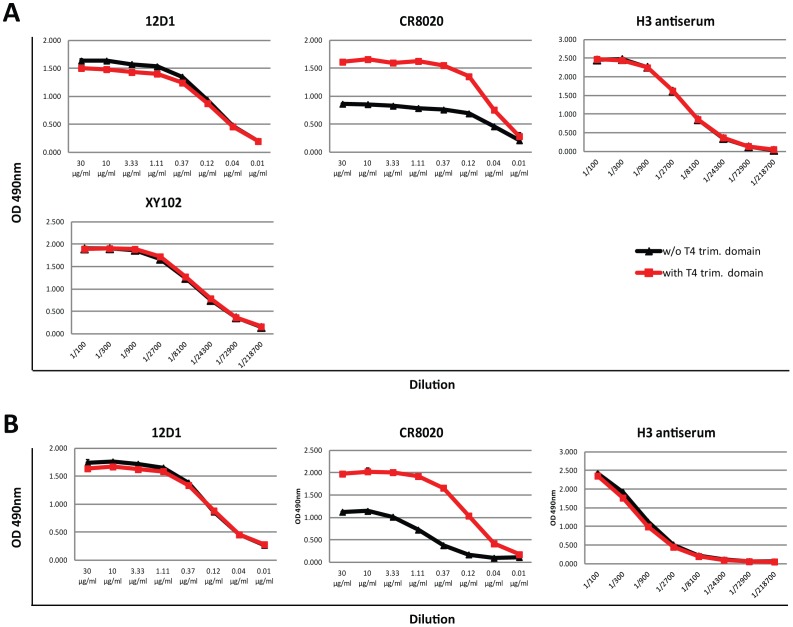
Binding of stalk-reactive antibodies to group 2 HAs. **A** Binding of stalk-reactive antibodies 12D1 and CR8020 and head-reactive antibody XY102 and H3 antiserum to recombinant soluble HK68 HA without (w/o T4 trim. domain, black lines) or with (w/T4 trim. domain, red line) trimerization domain. **B** Binding of stalk-reactive antibodies 12D1 and CR8020 and H3 antiserum to recombinant soluble Wisc05 HA without (w/o T4 trim. domain, black lines) or with (w/T4 trim. domain, red line) trimerization domain.

For group 2 HA-binding antibodies, however, a different pattern emerged. In order to test the effects of a trimerization domain on reactivity of stalk antibodies with group 2 HAs, we used broadly reactive antibodies CR8020 and 12D1. CR8020 is known to bind a conformational epitope in group 2 HAs [5], while 12D1 is thought to bind to a linear epitope within the long alpha helix (LAH) of the HA2 subunit [8]. CR8020 binding to HK68 and Wisc05 HAs with trimerization domains was greatly enhanced over binding to HAs without trimerization domain (Figure 5A and B). However, lack of the trimerization domain did not completely abolish binding as seen with group 1 HAs. It seems, taking the data from our crosslinking assay into account, that H3 HAs are more efficient in forming trimers in the absence of a trimerization domain than group 1 HAs. Interestingly, 12D1 did not distinguish between HAs with or without the trimerization domain (Figure 5). Because it has been shown that 12D1 binds to the LAH alone, it is possible that its binding is less conformation dependent and therefore does not require a trimerized stalk domain at all. Again, control sera and anti-globular head antibody XY102 did not distinguish between the two different forms of HA substrates. Taken together, these data strongly suggests that stalk-reactive antibody binding to soluble/secreted HA substrates is greatly influenced and enhanced by a C-terminal trimerization domain and that this phenotype is stronger for group 1 than for group 2 reactive antibodies.

### Conformational Epitopes in the Stalk Domain are More Vulnerable to Destabilization by Freeze-thaw Cycles than Epitopes in the Globular Head Domain

**Figure 6:**
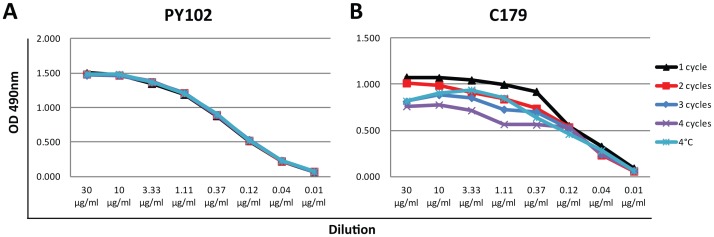
Stability of the C179 stalk epitope during storage and freeze-thaw cycles. PR8 HA with trimerization domain was stored at −80°C and subjected to one (1 cycle), two (2 cycles), three (3 cycles) or four (4 cycles) freeze-thaw cycles or stored at 4°C (4°C) for 60 days. **A** Reactivity of anti-globular head antibody PY102 or **B** stalk-reactive antibody C179.

Stability during storage is an important issue for reagents as well as vaccines. In order to assess stability of stalk epitopes on HA proteins with trimerization domains during storage, we chose the PR8 HA as a model. We stored purified protein at 4°C in a laboratory refrigerator for a period of 60 days or kept it at −80°C in a freezer, and subjected the protein to either one, two, or three freeze-thaw cycles. We then coated ELISA plates with the differentially treated proteins and assessed the integrity of relevant epitopes of the recombinant HA either with the stalk-reactive antibody C179 or with the head-reactive antibody PY102. As expected, the reactivity of C179 was inversely related to the number of freeze-thaw cycles the protein was subjected to (Figure 6). It is of note that long-term storage at 4°C also negatively affected C179 binding. In contrast, protein treatment or storage conditions did not affect the reactivity of head domain-reactive mAb PY102. These data are congruent with the binding phenotypes of stalk-specific antibodies to trimerized and non-trimerized forms of soluble HA protein, demonstrating the fragility of stalk epitopes compared to those of the head.

## Discussion

Secreted, soluble forms of HA can easily be produced in research laboratories using baculovirus- or mammalian cell-based expression systems. In this report, we investigated the influence of a C-terminal T4 trimerization domain on the binding of stalk-reactive broadly neutralizing antibodies to a secreted soluble form of HA. We expressed a variety of different group 1 and group 2 HAs and found that the addition of a C-terminal trimerization motif seems to increase the stability of these glycoproteins as trimers during expression and prevents both site-dependent as well as non-specific cleavage. Of note, we demonstrate that specific stalk-reactive antibody binding is largely dependent on trimerization of soluble HAs. Binding of stalk-reactive broadly neutralizing antibodies only occurs in the presence of a C-terminal trimerization domain for group 1 HAs. All tested mAbs bound exclusively to the HAs that had a trimerization domain; binding was completely abolished when a trimerization domain was not expressed. For group 2 HAs, a trimerization domain was not required for stalk-specific antibody binding. It is of note that while 12D1 binding was completely unaffected by trimerization domain expression, CR8020 binding was greatly enhanced when a trimerization domain was included in the construct. In contrast, antibodies directed against the globular head domain of both group 1 and group 2 HAs were not affected by the presence or absence of the trimerization motif. Our data therefore reflects a fundamental difference in terms of stalk- and head- specific antibody binding to soluble HA proteins. The above observances are also reflective of the intrinsic differences in the nature of the epitopes of group 1 and group 2 stalk-reactive antibodies. In the former, it was shown in crystal structure analysis that group 1 mAbs such as CR6261 and F10 bound to the hydrophobic groove of the stalk region formed between two monomers of the HA [4], [7]. Thus it is easy to envision that the epitopes of group 1 mAbs are highly dependent on correctly positioned monomers in a trimerized HA. The described epitope of group 2 mAb CR8020 is located on the outer surface of the five-stranded beta sheet and the C-terminal region of the fusion peptide of an HA monomer [5]. This fact could explain residual binding of CR8020 to monomers of the HA. However, the addition of a trimerization domain greatly enhanced CR8020 binding, suggesting that binding to this epitope is facilitated if the monomer is incorporated in a correctly folded trimer. Additionally, we have shown in our own studies that mAb 12D1 can solely target the LAH of the H3 stalk [8]. The latter two group 2 epitopes are not as highly dependent on trimerized HA as the group 1 epitopes as also reflected in our ELISA binding assays.

Our work also highlights differences between group 1 and group 2 soluble HAs. Group 1 HAs that have a C-terminal trimerization domain form uniform trimers whereas group 1 HAs lacking a trimerization domain form high molecular weight multimers. This multimerization of group 1 HAs that do not express a trimerization domain is most likely mediated by a multimerization motif present on the stalk domain of the HA1 subunit which is also functional when the HA1 subunit is expressed without the HA2 subunit [34]. This multimer formation was recently described for H1 and H5 HAs [34]. We suggest that the process of multimerization is not sufficient to stabilize conformational epitopes in the stalk domain. Group 2 HAs, however, behave differently and seem to form mostly uniform trimers regardless of the presence or absence of a trimerization domain.

Trimerization domains have previously been described for use in influenza virus HA expression. The trimerization domain (foldon) of T4 phage fibritin, a trimeric beta hairpin propeller, was first used in crystallization studies of the 1918 H1N1 HA [23]; a leucine zipper trimerization motif derived from the yeast transcription activator GCN4 has also been used [14], [16], [17]. Here we demonstrate that the T4 trimerization domain allows for successful trimerization of soluble HA molecules and greatly increases the stability of these molecules following baculovirus expression. These findings are in line with previously published reports that demonstrate the enhanced immunogenicity of soluble influenza virus HAs that express a C-terminal trimerization domain [14], [15], though we are the first to report the effect of such stabilization techniques on stalk-antibody binding.

It is of interest that stalk-reactive antibodies were crystallized with trimeric, recombinant HA proteins after the trimerization domain was removed [4], [7]. We therefore suggest that the trimerization domain is important for the initial formation of HA trimers in the endoplasmatic reticulum, but is not necessarily required once the trimer has formed. In conclusion, our results highlight the importance of a trimerization domain on soluble, secreted HAs for the detection and analysis of stalk-reactive monoclonal antibodies, as well as serological studies of animal or human sera.
